# Significance Testing and Multivariate Analysis of Datasets from Surface Plasmon Resonance and Surface Acoustic Wave Biosensors: Prediction and Assay Validation for Surface Binding of Large Analytes

**DOI:** 10.3390/s18103541

**Published:** 2018-10-19

**Authors:** Mihaela Puiu, Lucian-Gabriel Zamfir, Valentin Buiculescu, Angela Baracu, Cristina Mitrea, Camelia Bala

**Affiliations:** 1 R&D Center LaborQ, University of Bucharest, 4-12 Regina Elisabeta Blvd., Bucharest 030018, Romania; elenamihaela.puiu@g.unibuc.ro (M.P.); lucian-gabriel.zamfir@cdi.unibuc.ro (L.-G.Z.); 2 ICUB, University of Bucharest, 36-46 M. Kogalniceanu Blvd., Bucharest 050107, Romania; 3 National Institute for Research and Development in Microtechnologies—IMT Bucharest, 126A Erou Iancu Nicolae Street, Voluntari 077190, Ilfov, Romania; valentin.buiculescu@imt.ro (V.B.); angela.baracu@imt.ro (A.B.); 4 S.C ROM-QUARTZ S.A, 126A Erou Iancu Nicolae Street, Voluntari 077190, Ilfov, Romania; cmitrea@minatech.ro; 5 Department of Analytical Chemistry, University of Bucharest, 4-12 Regina Elisabeta Blvd., Bucharest 030018, Romania

**Keywords:** surface acoustic wave, surface plasmon resonance, aflatoxin B1, significance testing

## Abstract

In this study, we performed uni- and multivariate data analysis on the extended binding curves of several affinity pairs: immobilized acetylcholinesterase (AChE)/bioconjugates of aflatoxin B_1_(AFB_1_) and immobilized anti-AFB_1_ monoclonal antibody/AFB_1_-protein carriers. The binding curves were recorded on three mass sensitive cells operating in batch configurations: one commercial surface plasmon resonance (SPR) sensor and two custom-made Love wave surface-acoustic wave (LW-SAW) sensors. We obtained 3D plots depicting the time-evolution of the sensor response as a function of analyte concentration using real-time SPR binding sensograms. These “calibration” surfaces exploited the transient periods of the extended kinetic curves, prior to equilibrium, creating a “fingerprint” for each analyte, in considerably shortened time frames compared to the conventional 2D calibration plots. The custom-made SAW sensors operating in different experimental conditions allowed the detection of AFB_1_-protein carrier in the nanomolar range. Subsequent statistical significance tests were performed on unpaired data sets to validate the custom-made LW-SAW sensors.

## 1. Introduction

Affinity sensors using gold/thiol chemistry for immobilization of biomolecules onto surfaces are probably the most versatile platforms for real time monitoring of biomolecular events through molecular recognition [1]. Due to their inherent properties, thin gold films are perfectly suited to detect a broad range of analytes using surface plasmon resonance and surface acoustic wave sensors, supporting both functionalization with organic self-assembled layers and interfacing of electrochemical, optical or piezoelectric transducers [2]. During the past decade, surface acoustic wave (SAW) sensors have emerged as alternative to surface plasmon resonance devices, owing their enhanced sensitivity to changes in mass, density and viscosity near the surface [3]. SPR is an optic phenomena following the interaction between an incident photon stream and the charge density waves travelling along a metallic surface, usually gold (designated as “surface polaritrons”) [2,4]. At settled conditions (angle, phase or wavelength) the energy of the incident photons is delivered to the surface polaritrons, causing a sharp decrease of the reflected beam intensity. The detection principle of the SPR devices lies in the changes of the refractive index due to the mass changes near the metallic surface [5]. At fixed wavelength the detection is achieved through the measurements of the angle shift at which the SPR effect take place (the so-called angle interrogation). On the other side, SAW devices use piezoelectric materials to generate an acoustic wave [3]. Love waves (LW) propagate near the surface of the piezoelectric material supporting shear horizontal waves, if it is laid on the top of the piezoelectric substrate and over the layer with a lower shear velocity [6]. The velocity and/or amplitude of the wave is strongly dependent on the medium contacting the surface [7]. In a typical LW-SAW approach an electrical signal is converted at interdigital transducers (IDT’s) into polarized transversal waves propagating parallel to the sensing surface, due to the piezoelectric properties of the substrate material [8]. The travelling wave is confined to an independent guiding layer and not to the piezoelectric substrate. Thus, the acoustic energy is concentrated mostly within the guiding layer and lesser in the bulk of the piezoelectric material. The waves are travelling across the sensitive area and are modified by biochemical events occurring at its surface [9]. Then, the wave is converted back at another IDT into an electrical signal. Input and output signals are transformed into a resulting signal of frequency or phase changes, which can then be correlated to the corresponding mass or mechanical properties in the liquid covering the sensitive surface of the sensor. Regardless of the nature of affinity pair, both SPR and SAW sensors are prone to baseline fluctuations due to the loss of activity of the immobilized molecules after a certain time span. Since the maximum binding capacity of the ligand decreases in time, new calibration plots are required and additional time-consuming steps involving costly reagents need to be performed [4]. On the other-hand, a “prequel” kinetic study may provide kinetic parameters depicting the time-evolution of the affinity interaction, which can be used further to predict the sensor’s response for the maximum binding capacity and to estimate the analyte concentration in pre-equilibrium conditions [10]. Therefore, we implemented several bio- and immunoaffinity pairs onto mass sensitive cells (SPR and SAW) operating in batch configurations. The fabricated LW-SAW sensors have the transducers covered with similar guiding layers but, due to their different piezoelectric substrates, they operate in different experimental conditions (i.e., sample volume and equilibration time span). Moreover, the analytical performances of the SAW sensors were optimized to suit the same binding assay as the commercial SPR sensor, in similar conditions. In this study we attempted to perform uni- and multivariate data analysis onto extended kinetic plots of affinity pairs such as acetylcholinesterase (AChE)/bioconjugates of aflatoxin B1(AFB1) and immobilized anti-AFB1 monoclonal antibody/AFB1-protein carriers. The aim is to obtain 3D plots depicting the time-evolution of the sensor response as a function of analyte concentration. These “calibration” surfaces may exploit the transient periods of the extended kinetic curves, prior equilibrium, creating a “fingerprint” for each analyte in a considerably shortened time frame compared to conventional 2D calibration plots.

## 2. Materials and Methods

### 2.1. Materials

#### 2.1.1. Chemicals

Reagent-grade chemicals: 11-mercaptoundecanoic acid (11-MUA), 1-ethyl-3-(3-dimethyl-aminopropyl carbodiimide) hydrochloride (EDC), N-hydroxysuccinimide (NHS) ethanolamine, absolute ethanol, sodium phosphate monobasic and dibasic, sodium chloride, sodium acetate, Tween 20, acetic- and hydrochloric acid were purchased from Merck (Kenilworth, NJ, USA). The piranha solution was a mixture 3:1 (*v*/*v*) of H_2_SO_4_ (purity > 95%, Fluka, Buchs, Switzerland) and H_2_O_2_ (30% *w*/*w*, Merck). Piranha solution is highly reactive and may cause injuries resulting from chemical and thermal burns if not handled with extreme caution. 

#### 2.1.2. Biomolecules for Acetylcholinesterase/AFB_1_-Bioconjugates Binding Assays

AChE from *Electrophorus electricus* (type VI, MW = 280,000, 222 IU·mg^−1^) and horseradish peroxidase (HRP) isoenzyme C (type VI, MW = 40,000, 233 IU·mg^−1^) were purchased from Sigma Aldrich (St. Louis, MO, USA) and used without any further purification. Protein concentrations for both enzymes were measured spectrophotometrically as it follows: AChE concentration at λ = 280 nm with a molar absorption coefficient ε = 125,730 cm^−1^ M^−1^ and HRP concentration at λ = 403 nm with ε = 102,000 cm^−1^ M^−1^. AFB_1_-HRP from *Aspergillus flavus* bioconjugate was obtained from R-Biopharm/Ridascreen (Darmstadt, Germany) for AFB_1_ detection. Bovine serum albumin (BSA) and AFB_1_-BSA bioconjugate from *Aspergillus flavus* were purchased from Sigma Aldrich. 

#### 2.1.3. Biomolecules for Anti-AFB_1_ Antibody/AFB_1_-Bioconjugates Binding Assays

AFB_1_ (2a) mouse monoclonal antibody (IgG) (MW = 150,000) was obtained from ABCAM (Cambridge, UK) as 1 mg/mL IgG solution in phosphate buffer saline (PBS) at pH 7.4 with 0.09% sodium azide. Bovine serum albumin (BSA) and AFB_1_−BSA bioconjugate from *Aspergillus flavus* were the same as in the previous ACHE/AFB_1_-BSA assay.

#### 2.1.4. Assay Buffers

The coupling buffer for both AChE and anti-AFB_1_ antibody in the SAW and SPR assays was 0.01 M acetate buffer solution (pH = 4.5) following the pre-concentration assays performed at several pHs, using SPR measurements. A 0.1 M phosphate buffer (PBS), pH 7.5, with 0.9% (*w*/*v*) NaCl and 0.05% (*v*/*v*) Tween 20 was used as association/dissociation buffer during the binding and washing steps in the AChE/AFB_1_-HRP and anti-AFB_1_ antibody/AFB_1_-BSA binding assays. 0.1 M HCl was used in the regeneration step. All aqueous solutions were prepared with ultrapure water, obtained with a Direct Q3 system (18.2 MΩ·cm, Millipore, Burlington, MA, USA).

### 2.2. Apparatus

The SPR measurements for immobilization and affinity studies were carried out with a double channel ESPRIT instrument (Metrohm-Autolab, Utrecht, Netherlands) under constant temperature (25 °C). The Autolab SPR chip, containing a 50-nm thick gold layer and an adhesive 5-nm titanium sublayer deposited on glass, was attached to the prism using an index-matching oil (the refractive index, n_d_^25°C^ being within 1.26–1.38). The experimental setup is based on a half-circular prism in the Kretschmann configuration.

The SPR angle shifts (Δθ_SPR_) for both channels (reference and sample) were measured in a batch regime, into 6 mm^2^ surface cells, at fixed wavelength (monochromatic, plane-polarized light source, λ = 670 nm). The change of the SPR signal was proportional with the amount of the bound protein, every 122 millidegrees (m°) angle shift corresponding to 1 ng·mm^−2^ of immobilized protein [11]; the baseline noise was 0.1 m° during a measurement time interval of 1 s. The 2D-binding curves were acquired and processed with the Metrohm-AUTOLAB Kinetic Evaluation 4.2.2 software. 3D plots and multivariate analysis were done with Table Curve 3D v.4.0 (Systat Software Inc., San Jose, CA, USA). The injection and the removal of the liquid samples from the SPR cuvette were performed with the micro-pumping system of the Autolab ESPRIT instrument, under automatic control. 

The measurements for antibody/antigen affinity binding and antibody immobilization were performed also with two SAW devices, each one containing a piezoelectric substrate, input and output interdigital transducers, a waveguide layer, and a sensitive layer together with the oscillating system.

#### 2.2.1. Configurations of the Custom-Made LW-SAW Cells

##### Piezoelectric Substrates for the LW-SAW Cells

**Figure 1:**
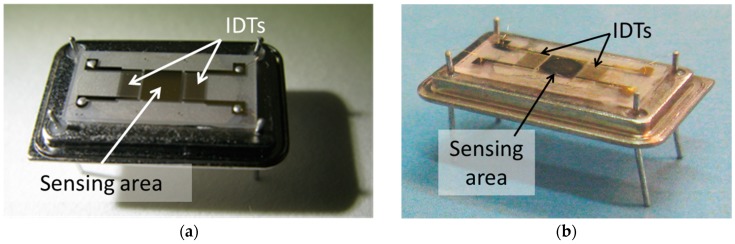
Custom-made LW-SAW chips fabricated on: (**a**) lithium tantalate and (**b**) ST-cut quartz crystal.

The LW-SAW sensors investigated in our study have been fabricated on two different piezoelectric substrates: quartz and lithium tantalate. A ST-cut Quartz (Roditi International Corporation Ltd., London, UK), was chosen as piezoelectric substrate material for the SAW-1 sensor, having a propagation velocity of 4996 m/s [12]. The SAW-2 device was fabricated on a piezoelectric lithium tantalate crystal (Roditi International Corporation Ltd., London, UK), Y-cut (36°), having a propagation velocity of 4118 m/s with respect to x-axis. For both devices, the shear horizontal mode surface acoustic wave interdigital transducers (IDTs) were designed and fabricated to operate in a delay line configuration for applications in liquid media. Each SAW chip consists of two identical IDT structures and a sensing area of 9 mm^2^ (Figure 1). The interdigital transducers have 50 fingers with a λ = 34 µm acoustic wavelength and a w = 8.5 µm finger width. The resulting central frequency was 140 MHz for SAW-1 and 121 MHz for SAW-2.

**Figure 2:**
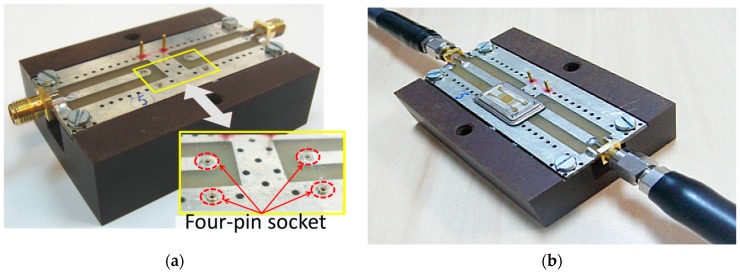
Fully assembled test fixture (**a**) Inset: close-up view of the four-pin sensor socket; (**b**) SAW sensor assembled on the test fixture.

Since the LW-SAW sensors have a transmission-type structure, a four-pin package was selected for the sensors’ chip assembly. Three pins were electrically isolated from the metallic base plate of the package by means of high-quality glass beads, while the remaining pin was inserted directly into the base plate. The pins were positioned perpendicularly to the package body, so that they can be inserted into a socket defined on a custom test fixture (Figure 2). 

The test fixture consists of a two-sided printed circuit board (PCB) supporting coplanar transmission lines (CPW) used for input and output signal exchange with the measurement system. The signal lines are accurately designed for broadband characterization of the packaged sensors. SMA connectors are available at both signal ports for connecting the test fixture to a laboratory grade vector network analyzer model 37397D from Anritsu (Morgan Hill, CA, USA), which covers full 40 MHz–65 GHz frequency range with its basic configuration.

##### Waveguide Mode and Deposition of the Sensitive Layers

The guiding layer has the additional role of shielding the IDTs, to prevent the corrosion of the metallic structures. The finger-pairs were fabricated by Electron Beam Evaporation (EBE-Temescal FC-2000, Livermore, CA, USA) of a 10 nm-thick Cr film (adhesion layer) and a 100 nm-thick gold layer, using a conventional lift-off photolithographic technique. The thickness of the metallic film (deposited by the evaporation method) [13] was accurately monitored through quartz crystal micro balance (QCM) measurements. The coating speeds (0.1 Å/s for the Cr adhesion layer and 3 Å/s for the Au layer) and the actual geometrical constraints were considered in real time during each deposition process. The 2 µm thick SiO_2_ guiding layer for both LW-SAW sensors was subsequently deposited via Plasma-enhanced chemical vapor deposition method (SPTS, Newport, UK). The aim of this layer was to increase the electromechanical coupling coefficient of the quartz substrate, to ensure electrical isolation and chemical protection for IDTs. At the end of the technological flow, a sensing area pattern (3 × 3 mm) was obtained using the same physical deposition technique (EBE) of Cr/Au films.

##### Design of the LW-SAW Sensor Testing Cells

Two testing cells from thermoplastic extruded polyamide (partially crystalline) were mounted onto the LW-SAW chips. These cells were provided with a cone-shaped hole which defined a limited zone for fluid samples of 6 mm^2^ surface. The liquid samples were injected into and removed from the cells with a micropipette. The measurement setup for the fabricated LW-SAW being broadband, we used the phase shift information to determine the analyte’s influence on the transmission coefficient. Thus, the interrogation mode of both LW-SAW sensors was phase shift (Δφ_SAW_).

### 2.3. Gold Surface Coating with a Thiolic Self-Assembled Monolayer 

The deposition protocol of an 11-mercaptoundecanoic acid (11-MUA) self-assembled monolayer (SAM) was used for both SPR and SAW assays. Prior to use the gold surfaces were activated by immersion in piranha solution (3:1 mixture of H_2_SO_4_ and H_2_O_2_) for 10 s; the gold surfaces were washed with ultrapure water and dried under a nitrogen stream. The cleaned surfaces were incubated with 1 mM solution of 11-MUA in absolute ethanol for 24 h, then rinsed with ethanol and ultrapure water for the removal of the residual 11-MUA, and finally dried by passing through a nitrogen stream. The SAM functionalized chips were then mounted onto SPR and SAW cells, respectively.

### 2.4. Immobilization of Biomolecules onto SAM Coated Surfaces

#### 2.4.1. Immobilization of AChE for SPR Binding Assays

The enzyme was immobilized via its primary amine groups through the EDC/NHS activation of the carboxyl residues from SAM [14]. The immobilization protocol was optimized in a previous work [10] to obtain a moderate binding capacity *R*_max_ of bound AChE (below 200 m°); this provided an AChE surface density high enough for affinity experiments involving high molecular weight (HMW) analytes, and low enough to avoid steric hindrance and mass transport limitation (MTL) effects. The optimized protocol consisted of the following steps:

- Activation of the carboxylic groups of immobilized 11-MUA via EDC/NHS esters
  This was achieved by successively injecting 50 µL of a 1:1 mixture of 0.4 M EDC/0.1 M, three times for 10 min;
- Optimization of the coupling buffer and of the concentration for AChE immobilization onto SAM.
  AChE solutions were prepared in a 10 mM acetate buffer (pH 4.5). The pH value was chosen below 1 unit of the isoelectric point of AChE (pI = 5.5) to avoid repulsive interactions between the negatively charged carboxyl groups of 11-MUA and the negatively charged enzyme; AChE was immobilized by injecting 50 µL of solution of 1 µM AChE (protein concentration) for 20 min;
- Blocking of the remaining active sites
  This step was achieved by injection of 50 µL of 1 M ethanolamine (pH 8.5) for 20 min.

All mentioned operations were followed by rinsing with a 0.1 M PBS solution (pH 7.5). 

#### 2.4.2. Immobilization of Anti-AFB_1_ Antibody for SPR and SAW Assays

The procedure of immobilization of the anti-AFB_1_ antibody onto thiolic SAM followed the same steps as described above for AChE. The optimum coupling buffer was also 10 mM acetate buffer (pH 4.5)—chosen after pre-concentration experiments at various pHs, from 3.5 to 6. The injection concentration was obtained through 1:1000 dilution of the stock solution and was 1 µg/mL.

### 2.5. Monitoring AChE/AFB1-HRP and AChE/AFB_1_-BSA Binding through SPR Measurements 

The AChE/AFB_1_-HRP and AFB_1_-BSA bio interactions were monitored following the injection of 50 µL of AFB_1_-HRP or AFB_1_-BSA solutions, prepared in their interaction buffers: 0.1 M PBS (pH 7.5) for AFB_1_-HRP and 0.1 M acetate buffer (pH 4.5) for AFB_1_-BSA. The coupling buffer for AFB_1_-BSA was chosen so that no electrostatic repulsions between AChE and BSA would occur, due to their acidic isoelectric points, 5.5 and 4.7, respectively. These solutions were kept in contact with the immobilized AChE for 40 min during the association phase, to reach the thermodynamic equilibrium. Then, the solution was drained out, and 50 µL PBS solution was injected for the dissociation phase. We used a double channel system, to extract any potential contributions to the SPR signal due to non-specific interactions between the protein part of the bioconjugate and SAM modified surface. Thus, AChE was immobilized in both sample and reference channels. Equal volumes of AFB_1_-HRP and HRP or AFB_1_-BSA and BSA of the same concentration were injected simultaneously onto sample and reference cells during the AChE/AFB_1_-HRP assays. Thus, the change of SPR response after subtracting the reference signal from the sample signal was assigned only to the binding of the AFB_1_ part of the bioconjugate to AChE. The concentrations of AFB_1_-HRP and AFB_1_-BSA ranged within 0.25 to 5 µM. Each binding experiment was followed by a dissociation step with 0.1 M PBS pH 7.5 (for AChE/AFB_1_-HRP binding) or 0.1 M acetate buffer (for AChE/AFB_1_-BSA binding) and a regeneration step with 0.1 M HCl incubated for 10 min over the sensor surface. All SPR measurements were performed at a constant temperature (25 °C).

### 2.6. Monitoring Anti-AFB_1_ Antibody/AFB_1_-BSA Interaction through SPR and LWSAW Measurements 

#### 2.6.1. Binding Protocol for SPR Cells Operating in Batch Configuration

The anti-AFB_1_ antibody/AFB_1_-BSA assay was performed as follows:

50 μL of AFB_1_-BSA in PBS solution (pH 7.5), with concentrations ranging from 3 to 265 nM were injected onto the chip surface. The solutions were kept in contact for 40 min to allow to the sensor’s response to equilibrate. Then, the solutions were drain out and 50 μL PBS were injected to start the dissociation phase. A double channel system was used, as in the case of AChE/AFB_1_-bioconjugate binding assay. The antibody was immobilized in both reference and sample cells. During the interaction, 50 μL of AFB_1_-BSA solution were injected in the sample cell, while 50 μL of BSA solution of the same concentration as the protein part of the bioconjugate were passed through the reference cell. Therefore, the increase of SPR signal after subtracting the reference signal from the sample was assigned only to the binding of the AFB_1_ part of the bioconjugate to the antibody epitope. Each binding experiment was followed by a dissociation step with 0.1 M PBS pH 7.5 and a regeneration step with 0.1 M HCl incubated for 10 min over the sensor surface. 

#### 2.6.2. Binding Protocol for LW-SAW Cells Operating in Batch Configuration

The optimized sample volume for the SAW-1 sensor (with quartz as piezoelectric substrate) was 5 μL, while 20 μL was the optimized sample volume for the SAW-2 sensor (with LiTaO_3_ as piezoelectric substrate). The concentration range for AFB_1_-BSA in PBS solutions (pH 7.5) was within 0.3–200 nM. Separate assays with BSA were performed as blank experiments and the SAW signal shift corresponding to BSA non-specific binding was extracted from the SAW signal shift corresponding AFB_1_-BSA. To avoid the time-consuming regeneration steps required after each binding assay, we used a successive additions format. Thus, the dissociation and the regeneration sequences were carried out after the stabilization of the SAW signal corresponding to the highest concentration of AFB_1_-BSA. After the addition of each sample, the sensors were left to equilibrate for 10 min prior to phase shift measurements. The liquid was removed with a micropipette afterwards, and the surface was gentle dried under a nitrogen stream. Then, a new addition of the sample followed. After the addition of the last sample, the experiment was followed by a dissociation step with 0.1 M PBS pH 7.5 and a regeneration step with 0.1 M HCl incubated for 10 min over the sensor surface. All SPR/SAW measurements of anti-AFB_1_ antibody/AFB_1_-BSA binding were performed at 25 °C.

## 3. Results and Discussion

### 3.1. Affinity and Kinetic Analysis of AChE/AFB_1_-HRP and AChE/AFB_1_-BSA Interactions

AChE/AFB_1_-HRP and AChE/AFB_1_-BSA were chosen as model systems for the binding kinetics/affinity of HMW analytes to univalent ligands. Such affinity pairs are frequently implemented onto various transducers for the direct detection of large analytes or in indirect detection of low molecular (LMW) weight compounds in direct/indirect competitive formats. AFB_1_ acts as non-competitive inhibitor of AChE, by reversibly binding at a *peripheral site* (P), located at the entrance of the active site of the enzyme [15]. It was previously reported that AChE /AFB_1_-HRP binding follows a Langmuir-like pattern characteristic for univalent ligand/univalent analyte interaction [10]. Therefore, AFB_1_-HRP was chosen as analyte in our study to estimate the maximum binding capacity of the immobilized AChE expressed as maximum equilibrium response of the SPR sensor (*R*_max_). For a bimolecular event involving molecules A (analyte) and L (immobilized ligand), resulting in a surface-bound complex AL, the equilibrium association constant (or affinity constant) *K_a_* and dissociation constant *K_d_* are given by Equations (1) and (2):

(1) $$K_{a}=\frac{{\left[AL\right]}_{eq}}{{\left[A\right]}_{eq}{\left[L\right]}_{eq}}\;(M^{-1})$$

(2) $$K_{d}=\frac{1}{K_{a}}\;(M)$$

The SPR responses, R and R_max_ are directly correlated with the molecular weight of the bound analyte, with the actual and maximum concentrations of the surface complexes [AB] and [AB]_max_, respectively; [AB]_max_ and [L]_0_ (the surface density of the active bound ligand) are equal only for 1:1 interaction (with all the binding sites being occupied). It was previously shown that no MTLs affect the AChE/AFB1-HRP interaction [10], and consequently, no significant analyte depletion occurs during the association phase and when the thermodynamic equilibrium is reached; therefore, one can approximate the injection concentration of the analyte, with the equilibrium concentration. One can define the normalized response of the sensor:

(3) $$R_{norm}=\frac{R}{R_{\max}}=\frac{\left[AL\right]}{{\left[L\right]}_{0}}$$

On the other hand, *K_a_* as a function of sensor response can be obtained by combining Equations (1) and (3) at equilibrium:

(4) $$K_{a}=\frac{R_{eq}}{{\left[A\right]}_{eq}\left(R_{\max}-R_{eq}\right)}$$

and finally, the dependence of the normalized SPR response at equilibrium on the injection concentration of the analyte can be expressed as:

(5) $$\frac{R_{eq}}{R_{\max}}=\frac{K_{a}{\left[A\right]}_{eq}}{1+K_{a}{\left[A\right]}_{eq}}$$

or:

(6) $$\frac{R_{eq}}{R_{\max}}=\frac{{\left[A\right]}_{eq}}{K_{D}+{\left[A\right]}_{eq}}$$

Equations (5) and (6) describe the 1:1 binding ligand/analyte according to the Langmuir pattern [3].

**Figure 3:**
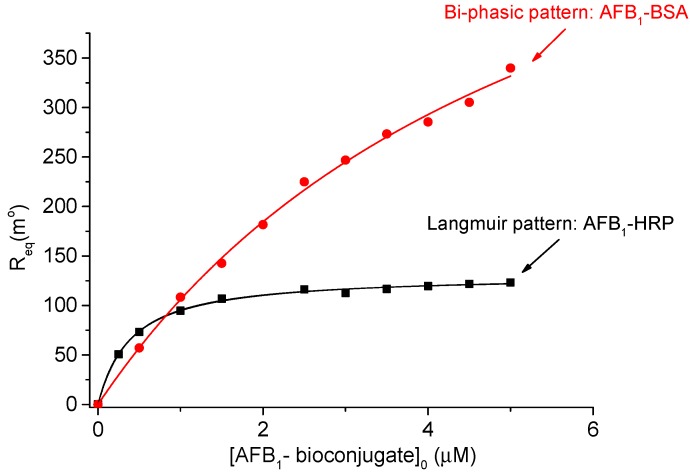
Different affinity patterns for AFB_1_-BSA/AChE and AFB_1_-HRP/AChE binding interaction after recording the SPR output at equilibrium (AChE surface density = 3.66 fmol·mm^−2^, T = 25 °C).

In our study, AChE was immobilized following the protocol depicted in the previous section. From the immobilization SPR sensograms we estimated the surface density of the bound AChE, obtained after the injection of 1 µM AChE, according to the equation:

(7) $${\left[L\right]}_{s}=\frac{R_{L}\cdot 10^{-9}}{122\cdot M_{L}}\;\mathrm{mol}\cdot {\mathrm{mm}}^{-2}$$

where [*L*]*_s_* is the surface density of the bound ligand, 122 is angle shift (in millidegrees) corresponding to 1 ng·mm^−2^ of immobilized protein [11], and *R_L_* the maximum SPR response recorded at the end of the immobilization procedure in PBS buffer. The estimated surface density of AChE obtained from three separate immobilization experiments was 3.66 ± 0.28 fmol·mm^−2^. We developed further the coupling experiments with AFB_1_-HRP, set as models for uni-univalent ligand/analyte interaction. The recorded SPR curves were zeroed on the y and x axes just prior to the start of the injection, and SPR signal from the reference channel was subtracted from the SPR signal corresponding to the sample channel. Since no significant MTL effects occurred during the binding assay, the equilibrium concentration of AFB1-HRP was approximated to the injection concentration. The SPR binding sensograms provided a maximum binding capacity (*R*_max_) at equilibrium of 125 m° with respect to AFB_1_-HRP. While the affinity plot SPR response vs. AFB1-HRP concentration confirmed Langmuir-like behavior, the AChE/AFB_1_-BSA displayed rather a different profile. The best-fit equation matching the SPR response vs. AFB_1_-BSA concentration was the bi-phasic model, corresponding to the following equilibria [16,17]:

(8) $$A\;+\;L\overset{k_{1on}}{\underset{k_{1off}}{⇌}}AL$$

(9) $$A\;+\;L*\overset{\;k_{2on}\;}{\underset{k_{2off}}{⇌}}AL*$$

characterized by the two sets of kinetic constants: association kinetic constants *k*_1*on*_ and *k*_2*on*_ and dissociation constants *k*_1*off*_ and *k*_2*off*_; L* is considered a less active form of ligand interacting with the analyte, having a lower affinity constant, *K_a_*_2_ [18]. In these conditions, the bi-phasic model [11] describes better the apparent dependence of the SPR response on the concentration of the injected AFB_1_-BSA (Equation (10), Figure 3):

(10) $$R_{eq}=R_{1}{}_{\max}\frac{K_{a1}{\left[A\right]}_{eq}}{K_{a1}{\left[A\right]}_{eq}+1}+R_{2}{}_{\max}\frac{K_{a2}{\left[A\right]}_{eq}}{K_{a2}{\left[A\right]}_{eq}+1}$$

where *R*_1max_ and *R*_2max_ are the maximum binding capacities of the ligand in its active and less active form, respectively. 

This particular variation of the SPR response at the binding of AFB_1_-BSA provided a linear range within 0.25–2 µM, at far more increased concentration values than at the binding of AFB_1_-HRP (0.008–0.032 μM [10]). The best-fit values of the parameters *R*_max1_, *R*_max2_, *K_a_*_1_ and *K_a_*_2_ from the bi-phasic model were estimated through non-linear regression analysis and were used further as input values in the multiple nonlinear regression algorithm applied on the real-time binding SPR sensograms. 

#### Prediction and Simulation of the SPR Response Using the Bi-Phasic Model

Both Langmuir and bi-phasic affinity curves can be used further to build calibration plots for the direct detection of the AFB_1_-bioconjugate detection (or for the indirect detection of AFB_1_ itself) by extracting the linear range from the extended curves. There are two main disadvantages of this approach, especially when the bi-phasic model matches the experimental curve: first, the experiments are time-consuming, since only the association phase lasts 40 min to reach equilibrium and second, there are relatively few points in the affinity plot and many parameters in the model, which cause large standard errors in the parameters’ estimates. To overcome these drawbacks, we attempted to exploit the transient parts from the binding sensograms, prior reaching equilibrium, and to build a 3D calibration plot using kinetic analysis. The kinetic equation depicting the time-evolution of the SPR output according to the bi-phasic model (when no MTLs are involved) is a sum of two exponential functions [11]:

(11) $$R_{t}=R_{1}{}_{\max}\frac{k_{1}{}_{on}[A]}{k_{1}{}_{on}[A]+k_{1}{}_{off}}\times \left(1-e^{-\left(k_{1}{}_{on}[A]+k_{1off}\right)t}\right)+R_{2}{}_{\max}\frac{k_{2}{}_{on}[A]}{k_{2}{}_{on}[A]+k_{2}{}_{off}}\times \left(1-e^{-\left(k_{2}{}_{on}[A]+k_{2off}\right)t}\right)$$

**Figure 4:**
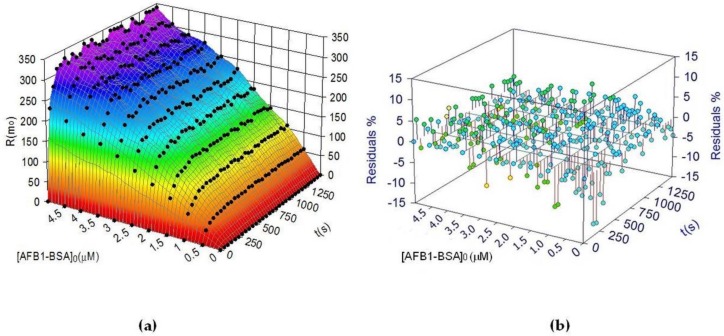
(**a**) 3D calibration plot obtained by fitting the bi-phasic model onto transient binding sensograms SPR response vs. time and vs. AFB_1_-BSA concentration (pH = 4.5, AChE surface density = 3.66 fmol·mm^−2^, T = 25 °C); (**b**) percentage residuals plot showing a random distribution of the residual points and a scattering range below 15%.

Here the dependent variable is the time-value of the SPR output R_t_, while the injection concentration of the analyte and the time span represent the independent variables. If this model matches the experimental data, then the experimental maximum response *R*_max_ for the same density of AChE as in the experiments with AFB_1_-BSA should be close to the sum of the estimated *R*_1max_ and *R*_2max_. Equation (11) was fitted on the 3D binding sensograms, exploiting the transient periods of the kinetic curves and using multivariate data analysis (nonlinear multiple regression) to validate the bi-phasic model characteristic to heterogenous ligand binding (Figure 4). 

The goodness of fit was assessed by the following topics: determination coefficient r^2^, standard errors in the parameter estimation and the residuals plot (Figure 4b) [19]. Moreover, the random distribution of the residual points and the low scattering range make a model error unlikely and confirms the bi-phasic pattern of AChE/AFB_1_-BSA binding. The best-fit parameters of the bi-phasic model are summarized in Table 1. Additional experiments were performed to check if the estimated *R_max_* matches the experimental value. A 50 µM AFB_1_-BSA provided an experimental *R_max_* of 530 m° while the estimated *R_max_* was 525 m°, confirming once more the validity of the chosen model.

The 3D plot was used further to estimate a sample concentration after recording the SPR output after less than 5 min, and the experimental concentration was compared to the predicted one (Table 2).

It can be noticed that the predicted concentrations at two different moments finally provided similar values. Therefore, we assume that the 3D calibration plots matched well the bi-phasic model. The binding capacities of the ligand can be estimated using several data points extracted from a single SPR sensogram and comparing them to the predicted values. Moreover, even if the ligand loses activity, the maximum binding capacity can be still estimated from a single sensogram at a settled concentration of analyte.

Apparently, the bi-phasic model describes enzyme/inhibitor interactions with relatively low affinity constants, when the immobilized enzyme either exists in multiple forms, or does not operate at its maximum activity. This type of analysis is more suitable for studying complex ligand/analyte biding and for predicting the activity loss of a bound ligand, rather than detecting analytes in the pico- or nanomolar range. Thereby, for a more accurate data acquisition we investigated a stronger paired interaction between AFB_1_-BSA and immobilized anti-AFB_1_ antibody on a SPR cell, then on two custom-made LW-SAW cells. 

### 3.2. Testing and Calibrating the LW-SAW Sensors Using Anti-AFB_1_ Antibody/AFB_1_-BSA Affinity Pair

**Figure 5:**
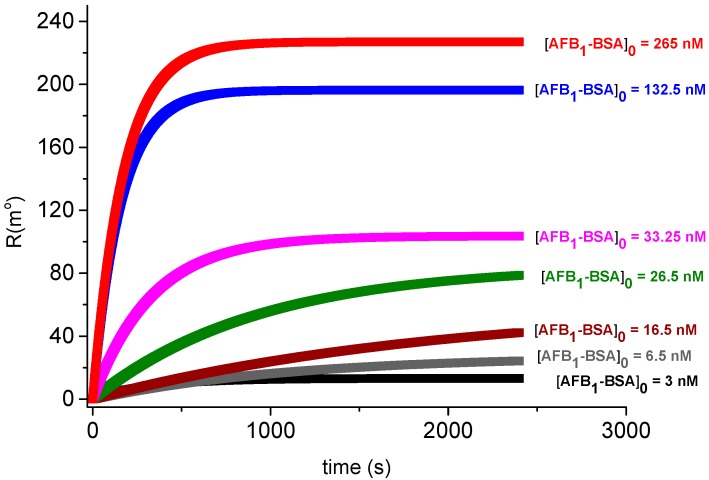
Real-time SPR binding sensograms for anti-AFB_1_ antibody/AFB_1_-BSA immunointeraction (pH = 7.5, *R*_max_ = 280 m°, T = 25 °C).

It was previously shown that the shape of the SPR response vs. AFB_1_-BSA concentration for the anti-AFB_1_ antibody/AFB_1_-BSA affinity interaction display a Langmuir pattern, characteristic for 1:1 ligand/analyte binding [3]. LW-SAW sensors may also display a hyperbolic dependence of the SAW response towards the concentration of the injected analyte, but the position of saturation zone in the LW-SAW assays differs significantly from the saturation region observed in the SPR assays. Therefore, the theoretical significance of the hyperbolic parameter can be assigned to the affinity constant of anti-AFB_1_ antibody/AFB_1_-BSA binding only in the case of SPR measurements. The real-time SPR binding sensograms recorded within 40 min provided the equilibrium responses for several AFB_1_-BSA concentrations (Figure 5).

**Figure 6:**
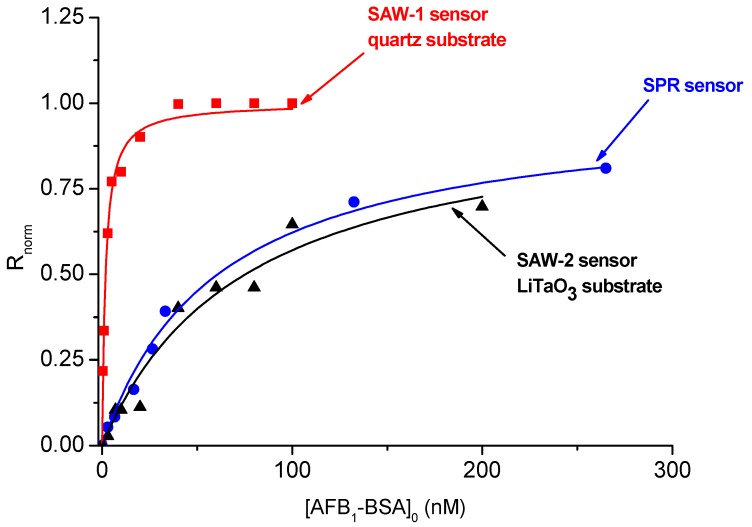
Langmuir-like behavior of the normalized response vs. injection concentration for the anti-AFB_1_ antibody/AFB_1_-BSA obtained with SPR and SAW-sensors (pH =7.5 and T = 25 °C).

Although these mass- sensitive cells operate in different conditions, it is of interest to check if they ultimately yield the same results and to compare their performance, since the immunoassay protocols are quite similar. One can expect an increased sensitivity of the LW-SAW sensors compared to the SPR sensor, since the LW-SAW detection is based on changes in mass, density and viscosity near the surface [6], and the SPR detection lies in changes in the refractive index due to mass variation near the surface [2,4]. Beside comparing the performance of SPR and LW-SAW sensors (sensitivity, limit of detection (LOD) and dynamic range) we also attempted to apply univariate data analysis (the Fisher’s ANOVA test and the *t*-test [20]) on samples with unknown concentration of AFB_1_-BSA. First, the normalized response from Equation (3) was plotted against the injection concentration of AFB_1_-BSA (Figure 6). A hyperbolic function was fitted on the binding curves obtained with the three sensors:

(12) $$y=\frac{P\cdot x}{1+P\cdot x}$$

where *y* is the normalized response of the sensor, *x* is the injection concentration of AFB_1_-BSA and *P* is the function parameter which can be assigned to the affinity constant *K_a_* only in the case of the SPR sensor.

It was noticed that SAW-1 sensor (based on a quartz piezoelectric substrate) display a significantly narrower linear range that both SPR and SAW-2 (based on a lithium tantalite piezoelectric substrate), while the SPR and SAW-2 affinity curves have similar shapes and dynamic ranges. We built the calibration lines for each sensor by extracting the linear parts of the affinity curves. The straight-line equations fitted on the experimental data *R_norm_* vs. AFB_1_-BSA concentration were used further to estimate the sensitivity (measured in normalized response units (n.r.u)/nM). LOD was defined as the concentration of AFB_1_-BSA corresponding to a normalized response equal to 10% of the saturation *R_norm_*. The results are summarized in Table 3.

### 3.3. Analysis and Validation of Data Using the F- and t-Tests for Unpaired Data 

We proceeded further with the Fisher’s ANOVA test to verify if the SPR and custom-made LW-SAW sensors display similar variances s^2^ (precision) for a significance level of 0.05. A two-tailed F-test was applied, since it was not expected that a sensor has a better precision that the other [20]. To do this, we prepared a sample with a concentration within the linear range of the sensors. This test was applied to data obtained with the commercial SPR sensor and the SAW-2, since the linear range of SAW-1 was below the linear range of the SPR and SAW-2 sensors. The test was performed on unpaired data (i.e., two sets of data consisting in results obtained using several samples drawn from a single source, usually with different number of replicates). The analyzed data are summarized in Table 4.

According to the null hypothesis (H_0_), there is no significant difference between two sample variances:

(13) $$H_{0}:S_{\mathrm{SPR}}^{2}=S_{\mathrm{SAW}-2}^{2}$$

According to the alternative hypothesis (H_A_), the difference between two sample variances is too large to be explained by indeterminate errors:

(14) $$H_{A}:{\;}_{\mathrm{SPR}}^{2}\neq S_{\mathrm{SAW}-2}^{2}$$

The experimental F parameter is:

(15) $$F_{\exp}=\frac{S_{\mathrm{SAW}-2}^{2}}{S_{\mathrm{SPR}-1}^{2}}=\frac{1.211}{0.972}=1.245$$

The calculated F_exp_ was subsequently compared to the critical value (5.19) of the statistical Fisher parameter, F_crit_ (0.05, 4, 5) (for a significance level of 0.05, 4 degrees of freedom for the SAW-2 sensor and 5 degrees of freedom for the SPR sensor). Since F_exp_ < F_crit_, the null hypothesis was accepted and the alternative hypothesis was rejected, meaning there is no difference between the precision of the two tested sensors. 

We performed further the t-test, designed to compare the mean values for sample concentrations detected with the SPR and SAW-2 sensor. To estimate the experimental value of the t parameter (t_exp_), we calculated first a pooled standard deviation:

(16) $$S_{\mathrm{pool}-}{}_{2}=\sqrt{\frac{(n_{\mathrm{SAW}-2}-1)\cdot S_{\mathrm{SAW}-2}^{2}+(n_{\mathrm{SPR}}-1)\cdot S_{\mathrm{SPR}}^{2}}{n_{\mathrm{SAW}-}{}_{2}+n_{\mathrm{SPR}}-2}}=1.038$$

The null hypothesis:

(17) $$H_{0}:{\bar{C}}_{\mathrm{AF}B_{1}-\mathrm{BSA}}^{\mathrm{SPR}}={\bar{C}}_{\mathrm{AF}B_{1}-\mathrm{BSA}}^{\mathrm{SAW}-2}$$

where ${\bar{C}}_{\mathrm{AF}B_{1}-\mathrm{BSA}}^{\mathrm{SPR}}$ and ${\bar{C}}_{\mathrm{AF}B_{1}-\mathrm{BSA}}^{\mathrm{SAW}-2}$ are the average concentrations of AFB_1_-BSA measured with the SPR and SAW-2, respectively.

The alternative hypothesis:

(18) $$H_{A}:{\bar{C}}_{\mathrm{AF}B_{1}-\mathrm{BSA}}^{\mathrm{SPR}}\neq {\bar{C}}_{\mathrm{AF}B_{1}-\mathrm{BSA}}^{\mathrm{SAW}-2}$$

The experimental parameter is:

(19) $$t_{\exp}=\frac{\left({\bar{C}}_{\mathrm{AF}B_{1}-\mathrm{BSA}}^{\mathrm{SAW}-2}-{\bar{C}}_{\mathrm{AF}B_{1}-\mathrm{BSA}}^{\mathrm{SPR}}\right)}{S_{\mathrm{poo}l_{2}\cdot }\sqrt{\frac{1}{n_{\mathrm{SAW}-2}}+\frac{1}{n_{\mathrm{SPR}}}}}=0.25$$

Since t_crit_ (0.05, 9) is 2.62, H_0_ is accepted and H_A_ is rejected. This result indicates there is no significant difference between the accuracy of the SPR and SAW-2 sensors towards the detection of AFB1-BSA using the anti-AFB_1_ antibody/AFB_1_-BSA affinity assay. Thus, the SAW-2 sensor display similar performance as the SPR sensor for the same assay but is more advantageous from the perspective of shortening the analysis time. On the other hand, the SAW-1 sensor exhibited a lower LOD than the one obtained with the commercial SPR sensor and an increased sensitivity compared to the SPR detection.

## 4. Conclusions

The assay time can be significantly reduced during SPR measurements if surface calibration plots using the transient periods of the binding sensograms are exploited. On the other hand, the LW-SAW built-up on a lithium tantalate piezoelectric substrate display similar performance characteristics as the commercial SPR sensor for the same affinity pair but has the advantage of operating at shortened equilibration time. The LW-SAW sensor with a quartz piezoelectric substrate displayed an increased LOD, a narrower linear range compared to the commercial SPR and required a significantly reduced sample volume. Therefore, we conclude that the latest is more suitable for the sensitive detection of large analytes, while the LW-SAW sensor with tantalate as piezoelectric substrate can be successfully used for affinity studies.

## Figures and Tables

**Table 1:** Best-fit parameters for bi-phasic model using multiple non-linear regression (n = 3).

*k*_1*on*_ (M^−1^ s^−1^)	*k*_1*off*_∙10^3^ (s^−1^)	*k*_2*on*_ (M^−1^ s^−1^)	*k*_2*off*_ ∙10^3^ (s^−1^)	*R*_1max_ (m°)	*R*_2max_ (m°)	*R*_max estim_ (m°)	*R*_max exp_ (m°)	r^2^
2998 ± 248	9.65 ± 0.61	1785 ± 121	6.54 ± 0.51	380 ± 22	145 ± 10	525 ± 32	530 ± 29	0.9972

**Table 2:** Experimental and predicted sample concentration using the “calibration surface” (n = 3).

[AFB_1_-BSA] (M) Injection	Time (s)	*R*_exp_ (m°)	[AFB_1_-BSA] (M) Predicted
3.325 × 10^−7^	250	102 ± 9.1	(3.39 ± 0.24) × 10^−7^
3.325 × 10^−7^	300	120 ± 9.5	(3.401 ± 0.26) × 10^−7^

**Table 3:** Performance characteristics of the LW-SAW and SPR sensors for AFB_1_-BSA detection using an affinity assay (n = 3).

Sensor/Interrogation Mode	Parameter	r^2^	Linear Range (nM)	Sensitivity (n.r.u/nM)	LOD (nM)
SAW-1/Phase	0.5738 ± 0.048	0.9897	0.3–4	0.1491 ± 0.0101	0.2
SAW-2/Phase	0.0133 ± 0.0012	0.9627	5–30	0.0093 ± 0.0011	8
SPR /Angle	0.01645 ± 0.00101	0.9917	5–30	0.01033 ± 0.00016	10

**Table 4:** Unpaired data obtained with the SPR and SAW-2 sensors using the anti-AFB1 antibody/AFB1-BSA affinity pair.

Sensor	Average *R_norm_*	Average Concentration of AFB_1_-BSA (nM)	Variance (s^2^) (nM)	No. of Replicates (n)
SAW-2—phase interrogation	0.118	10.14	1.211	5
SPR—angle interrogation	0.148	9.98	0.972	6
